# Regional impacts of climate change and its relevance to human evolution

**DOI:** 10.1017/ehs.2020.56

**Published:** 2020-10-28

**Authors:** María Fernanda Sánchez Goñi

**Affiliations:** EPHE, PSL University, and University of Bordeaux, EPOC, UMR 5805, F-33615 Pessac, France

**Keywords:** Middle and Upper Pleistocene, Dansgaard–Oeschger cycles, Heinrich events, Neanderthal–Denisovan, *Homo sapiens*

## Abstract

The traditional concept of long and gradual, glacial–interglacial climate changes during the Quaternary has been challenged since the 1980s. High temporal resolution analysis of marine, terrestrial and ice geological archives has identified rapid, millennial- to centennial-scale, and large-amplitude climatic cycles throughout the last few million years. These changes were global but have had contrasting regional impacts on the terrestrial and marine ecosystems, with in some cases strong changes in the high latitudes of both hemispheres but muted changes elsewhere. Such a regionalization has produced environmental barriers and corridors that have probably triggered niche contractions/expansions of hominin populations living in Eurasia and Africa. This article reviews the long- and short-timescale ecosystem changes that have punctuated the last few million years, paying particular attention to the environments of the last 650,000 years, which have witnessed key events in the evolution of our lineage in Africa and Eurasia. This review highlights, for the first time, a contemporaneity between the split between Denisovan and Neanderthals, at ~650–400 ka, and the strong Eurasian ice-sheet expansion down to the Black Sea. This ice expansion could form an ice barrier between Europe and Asia that may have triggered the genetic drift between these two populations.

## Graphical abstract

**Figure:**
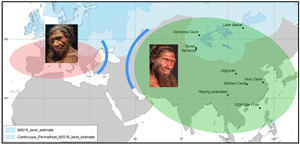

**Media summary:** Climate-driven ecosystem shifts created barriers and corridors affecting human biological and cultural evolution.

## Introduction

Long-term climate changes have been proposed as one of the main driving mechanisms of speciation, extinction, adaptation and changes in the distribution of animals and plants (Darwin, 1859; Gulick, 1872; Vrba, 1995; Cowling et al., 2008). Climate change would create new ecosystems where the best adapted species would survive (natural selection through competition; Darwin, 1859). Alternatively, climate change would produce either barriers or corridors favouring vicariance, i.e. the formation of small population through fragmentation or dispersal, and therefore genetic drift and natural selection (Gulick, 1872). Following the first mechanism, it has been proposed (deMenocal, 2011) that during the Quaternary, starting at ~2.58 million years ago (Ma), periods of dryness in Africa coinciding with the largest northern hemisphere glaciations led to the appearance of new morphological traits in grassland bovid species, and hominin speciation and extinction and, particularly, the emergence in Africa of the small-brained *Homo* (*H. erectus*) at ~2 Ma. Furthermore, the pervasive orbitally driven growth and decay of continental ice-sheets during the Quaternary resulted in sea-level changes and consequent shifts in the configuration of the continents. The recurrent emergence of land bridges and continental shelves during glacial periods provided migration routes and dwelling surfaces, and consequent species admixture between Africa and Eurasia (Vrba, 1995). Large ice-sheet development would also isolate populations, triggering vicariance in fauna, including hominin populations and plant communities, leading to genetic drift and speciation (Vrba, 1995). Vrba's ‘turnover pulse’ hypothesis stresses therefore large-scale climate changes as the forcing for speciation, extinction and migration events resulting in profound changes in species competition. However, an alternative hypothesis, the ‘variability selection’ hypothesis, identifies the increasing climatic variability recorded over the past million years as the trigger for increasing plasticity leading to speciation and population spread (Potts, 1998). Still other authors put forward the ‘pulsed climate variability’ hypothesis that combines the two former ones. They propose that precessional or half-precessional pulses of wetter and more variable conditions in Eastern Africa modified plant and faunal distribution patterns that would have created refugium zones and vicariance (Maslin et al., 2014).

Since the 1980s, the identification of the rapid, millennial to centennial, suborbital climatic variability punctuating the Quaternary Period has further led to questioning of the impact of these rapid climatic shifts on the biological and cultural human evolution. Different hypotheses have emerged linking climate changes with those in the geographic distribution of the fauna and flora, demography, genetic diversity and cultural evolution. Fragmentation and interaction between heterogeneous biomes, owing to climate changes in Africa over time, have been recently advanced to explain the isolation and admixture of human populations leading to the multiregional origin of *H. sapiens* ~300 ka (Hublin et al., 2017) and its biological and cultural evolution (Blome et al., 2012; d’Errico et al., 2020; Scerri et al., 2018; Will et al., 2019). In Europe, the replacement of Neanderthals by *H. sapiens* has been explained by a competition for the same ecological niches associated with the afforestation of the Iberian Peninsula in response to the rapid warming at *~*38 ka (Banks et al., 2008; d’Errico & Sánchez Goñi, 2003). In contrast, the millennial-scale changes seem to have had only a limited impact on the dispersal of *H. sapiens* towards Eurasia (Timmermann & Friedrich, 2016). The migration ‘out of Africa’ is explained by recurrent orbitally paced pluvial periods creating vegetated corridors from north-eastern Africa into the Arabian Peninsula and the Levant between 120 and 50 ka (Osborne et al., 2008; Timmermann & Friedrich, 2016). However, while habitat suitability for animals (including humans) would increase during wet periods in northern Africa, for example the ‘green Sahara’ episodes, the dispersal of *H. sapiens* from East Africa could be favoured by drying periods triggering forest fragmentation and the appearance of corridors in the heavily forested eastern and tropical regions (Blome et al., 2012).

The increasing climate variability and its amplitude, leading to larger and more recurrent ecological shifts, have been additionally advocated to explain the biological shaping of the African fauna and our ancestors (Caley et al., 2018; deMenocal, 2011; Krapp et al., 2019). It has also been argued that the most stable environments resulted in fewer innovations and less complex technology than those affected by cumulative high climatic variability (Richerson & Boyd, 2013). The high-frequency climatic variability would produce the development of new techno-complexes but the events would have been too short to trigger morphological changes in *H. sapiens* in response to the rapidly changing environments (Richerson et al., 2005).

**Figure 1.**
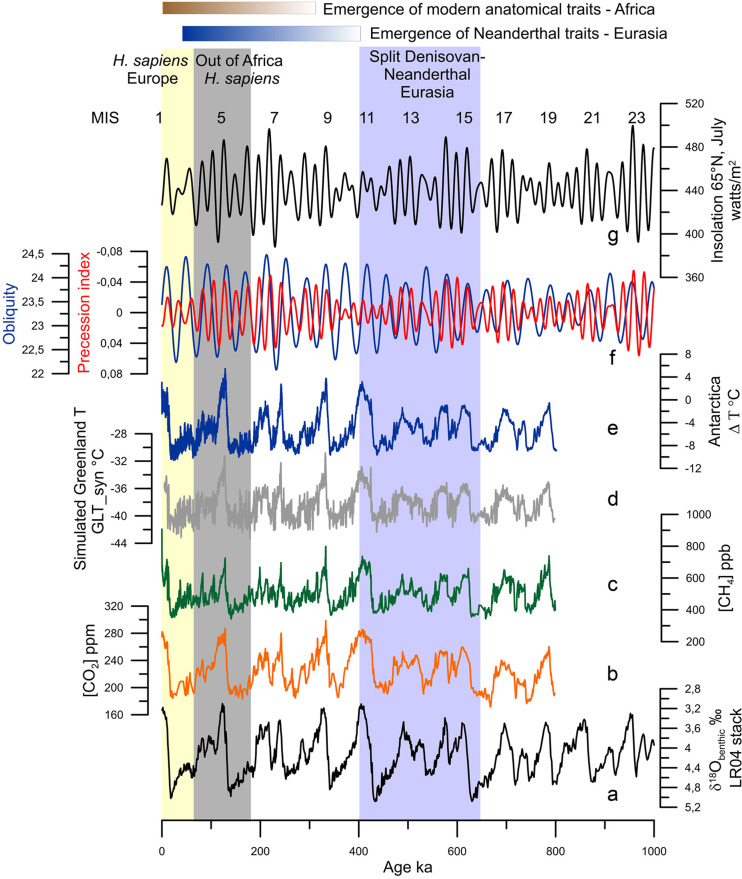
Orbital and long-term climatic changes over the last million years: (a) δ^18^O benthic foraminiferal LR04 stack record (Lisiecki & Raymo, 2005). (b) CO_2_ concentrations (Luthi et al., 2008). (c) CH_4_ concentrations (Loulergue et al., 2008). (d) Simulated Greenland temperatures (Barker et al., 2011). (e) Antarctic temperature anomalies (Jouzel et al., 2007). (f) Precession index (red) and obliquity (blue; Berger & Loutre, 1991). (g) Insolation variations in July at 65°N (Berger & Loutre, 1991). MIS: Marine Isotope Stage.

**Figure 2.**
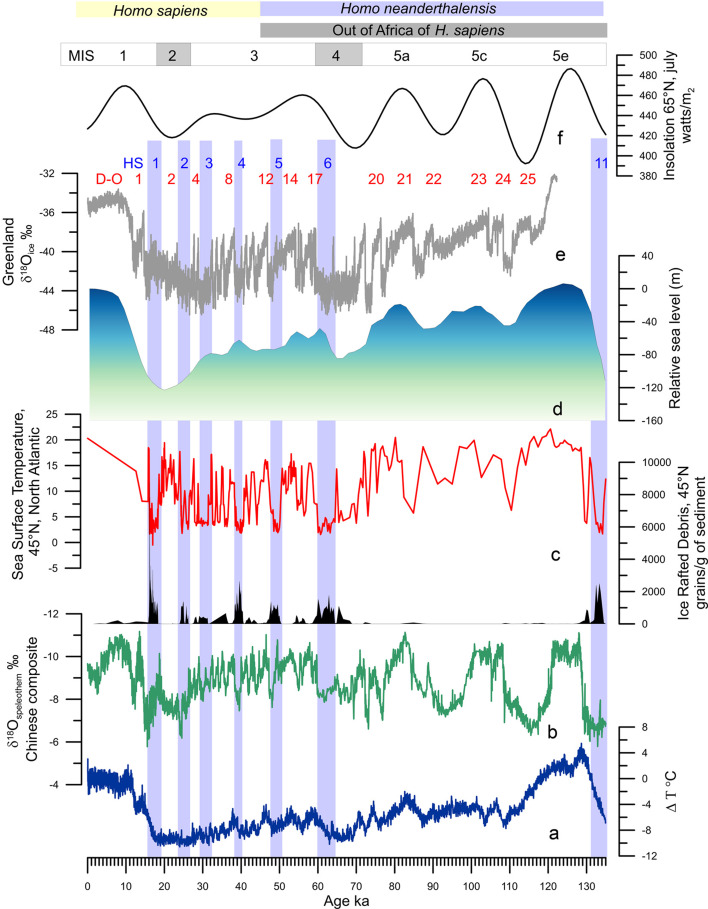
Millennial-scale climatic variability of the last climatic cycle. (a) Antarctic temperature anomalies (Jouzel et al., 2007). (b) Composite Chinese speleothem record (Cheng et al., 2016). (c) Ice-rafted debris from core MD04-2845 (45°N, 5°W) and Sea Surface Temperature record from foraminifera assemblages of core MD04-2845 indicating the position of HS 11 and HSs 6 to 1 (light blue bands; Sanchez Goñi et al., 2008 and unpublished data). (d) Relative sea-level changes (Waelbroeck et al., 2002). (e) Greenland ice core *δ*^18^O record (Rasmussen et al., 2014) indicating the Dansgaard–Oeschger (D–O) warming events. (f) Insolation variations in July at 65°N (Berger & Loutre, 1991).

This review aims to provide, based on relevant palaeoclimatic records, a comprehensive overview of the nature, timing and frequency of the African and Eurasian environmental changes that have characterized the past 1 million years (Myr). This period has witnessed key biological and cultural events in human evolution such as the increasing brain size and development of innovations in *Homo* (d’Errico et al., 2006; Richerson et al., 2005; Richerson & Boyd, 2013), the split between Neanderthals and Denisovan in Eurasia between 650 ka and 400 ka (Prüfer et al., 2017; Reich et al., 2010), the emergence of *H. sapiens* ~300 ka in Africa (Hublin et al., 2017) and their recurrent migration waves towards Eurasia (Beyer et al., 2020; Hublin et al., 2017), and the replacement of Neanderthals by *H. sapiens* in Europe ~36 ka (d’Errico & Banks, 2015; Figures 1 and 2). First, a synthesis of the global orbital and suborbital climatic variability over the last 1 Myr is presented, followed by a section that documents the impact of these climate changes on African and Eurasian ecosystems. Preferentially pollen-based vegetation records are used from which we can roughly estimate the available biomass resources for animals and humans. By comparing palaeoenvironmental changes with biological and cultural changes in Africa and Eurasia, the final section discusses the potential causality between climate changes and human evolution. In particular, the following points are addressed: (a) the multiregional emergence of *H. sapiens* and its dispersal out of Africa; (b) the emergence of Neanderthals at ~650–400 ka; (c) the replacement of Neanderthals by *H. sapiens* ~36 ka; and (d) the mechanisms triggering new technical strategies and complex societies during the Upper Palaeolithic.

## Climate changes during the last one million years

### Long-term orbitally driven climate changes

The glacial–interglacial cycles of the Quaternary, first revealed by the advances and retreats of northern hemisphere high-latitude ice-sheets and mountain glaciers (Agassiz, 1840), are fundamentally forced by changes in insolation (Milankovitch, 1941). Insolation is defined as the amount of energy per surface unit that the Earth receives from the Sun. Insolation is controlled by the distance between the Earth and the Sun that depends on eccentricity (the shape of the Earth's orbit), obliquity (the tilt of the Earth's axis) and precession (the orientation of the Earth's axis); the latter determines the amplitude of the seasons. These orbital parameters vary over time and trigger climatic variations occurring with quasi-periodicities lying between tens and hundreds of thousands of years (Berger & Loutre, 2004; Figure 1a, f, g). A change in insolation affects the Earth's five main climatic reservoirs – atmosphere (temperature, precipitation and greenhouse gas (GHG) concentrations), ocean, land surfaces, cryosphere and vegetation – and each of them affects, in turn, the Earth's other reservoirs through feedback mechanisms that amplify or reduce the original climate change, its frequency and duration (Ruddiman, 2001). The analysis of the oxygen isotopic ratio of benthic foraminifera preserved in deep-sea sedimentary sequences, an indicator of global ice volume, confirmed the Milankovich theory of the Ice Ages (Shackleton & Opdyke, 1973), and reveals that between 2.58 and *~*1 Ma, glacial–interglacial periods evolved with a 41,000 year cyclicity and shifted during the Middle Pleistocene Transition (MPT, 1.2–0.7 Ma) to a quasi-100,000-year ice-age cyclicity without any significant change in insolation (Mudelsee & Stattegger, 1997; Figure 1a). This means that the Quaternary was marked by global and large long-term climatic changes with progressively longer and stronger glacial periods towards the present (Lisiecki & Raymo, 2005).

Ten glacial–interglacial cycles, represented by 20 Marine Isotope Stages (MIS) from MIS 20 to MIS 1 (including the present interglacial), characterized the climate of the last 1 Myr (Lisiecki & Raymo, 2005). Odd and even numbers broadly correspond to interglacial and glacial periods, respectively. MIS 19 to MIS 5, ~781–126 ka, represent the Middle Pleistocene while the Upper Pleistocene encompasses the interval 126–11.7 ka, i.e. MIS 5 to the onset of the Holocene within MIS 1 (Cohen et al., 2013). Within interglacials, an alternation of three or five warm and cold substages can be identified, related with ice-volume minima during substages ‘a’, ‘c’ and ‘e’, and maxima, substages ‘d’ and b’ (Railsback et al., 2015). Excluding MISs 17, 13 and 7, all the interglacials experienced their warmest conditions at the beginning, just after the deglaciation concomitant with the highest sea level (Railsback et al., 2015; Figure 1d, e). The amplitude of glacial–interglacials before 450 ka were weaker compared with that after 450 ka, with MIS 19–13 interglacials cooler and marked by lower GHG concentrations than those from MIS 11 to MIS 1 (Yin & Berger, 2012). This glacial–interglacial change in amplitude is known as the Mid-Brunhes Event (MBE), and the causes are still unknown (Bouttes et al., 2020). As a consequence of the combined forcing of insolation and GHG concentrations (Figure 1b, c), climatic models indicate that MIS 5e (~126 ka), MIS 9e (~325 ka) and MIS 11c (~420 ka) were the warmest at the highest latitudes, associated with the strongest melting of ice-sheets (Yin & Berger, 2012, 2015), and data roughly confirm that it is the case (Pages, 2016). The durations of the interglacials also differ one from another, varying between 30,000 and 10,000 years with the longest being MIS 11c and the shortest MIS 7e (Pages, 2016).

### Suborbital, millennial to centennial, climate changes

Geological archives have shown that glacial–interglacial cycles have been punctuated by suborbital, millennial to centennial, climate changes (Jouzel et al., 2007; McManus et al., 1999) but their origins are still hotly debated (Alvarez-Solas & Ramstein, 2011; Bond et al., 1999). These archives further show that millennial-scale changes have affected all of the world's regions to a greater or lesser intensity and frequency: sea-surface temperatures in all of the oceans (Martrat et al., 2007; Voelker & participants, 2002), Asian monsoon (Cheng et al., 2016; Li et al., 2019), as well as air temperatures over Greenland (Barker et al., 2011; North Greenland Ice-Core Project (NorthGRIP), 2004) and Antarctica (EPICA, 2004; Figure 1d, e). These changes were generally associated with changes in ice volume (Siddall et al., 2003), GHG concentrations (Loulergue et al., 2008; Luthi et al., 2008; Spahni et al., 2005) and the intensity of oceanic circulation (Lynch-Stieglitz, 2017; Oppo et al., 2006). Millennial- to centennial-scale climatic shifts occurred therefore independently of the boundary conditions i.e. in glacial and interglacial periods, but their magnitude and frequency were larger when ice caps reached a critical mass, i.e. intermediate ice volume between interglacial and glacial conditions (McManus et al., 1999), such as during the middle part of the last glacial period or MIS 3 (~60–27 ka; Bond et al., 1999; Figure 2a–e).

The first evidence of the millennial-scale climate variability was detected in the δ^18^O of the Greenland ice cores during the last glacial period (MIS 5d-a, MIS 4, MIS 3 and MIS 2, i.e. ~115–14.7 ka; Dansgaard et al., 1984). It is so far the best documented suborbital climate variability and marked by a succession of regional warming–cooling events called Dansgaard–Oeschger (D–O) cycles (Figure 2e). These cycles usually lasted 500–2,000 years (Dansgaard et al., 1984) and were characterized by a large (7–16°C) and rapid (within a few decades) warming event followed by a progressive decrease in temperature and a final abrupt cooling (Wolff et al., 2010). The warming event and the progressive cooling phase form the Greenland Interstadial (GI), and the final cooling event leading to the cold phase form the Greenland Stadial (GS). The GI phases lasted between 100 and 2,600 years (Rasmussen et al., 2014; Wolff et al., 2010). The last glacial period was also punctuated by massive and repeated iceberg discharges, every 7,000–10,000 years, from the Laurentide Ice Sheet. These Heinrich events (HE) as they are called, substantially cooled the surface of the North Atlantic (Bond et al., 1993; Heinrich, 1988; Figure 2c, d). An HE is defined as the event synchronous with the deposition of the ice-rafted debris in the North Atlantic, preferentially between 45 and 50°N, resulting from the melting of the icebergs. In contrast the Heinrich Stadial (HS) is the cold phase associated with the HE that can last up to 3,000 years (Sanchez Goñi & Harrison, 2010). Weaker discharges with higher frequency than the HE resulted from the iceberg fragmentation from the British–Icelandic–Scandinavian ice cap (Elliot et al., 2001). Iceberg discharge has been simulated to last between 50 and 200 years (Roche et al., 2004). Some GSs encompass the HSs, while the others are associated with the British–Icelandic–Scandinavian minor iceberg discharges. The cooling events are related to decreases in the AMOC (Henry et al., 2016; Lynch-Stieglitz, 2017), but the cause of the ice-sheet collapse remains a subject of debate (Alvarez-Solas & Ramstein, 2011; Barker et al., 2015). Since the identification of these millennial-scale climate changes in the atmosphere of Greenland, D–O cycles, and in the North Atlantic Ocean, HEs, the terrestrial palaeoclimatic community has focused its efforts on investigating the regional expression of this variability (e.g. Fleitmann et al., 2009; Sanchez Goñi et al., 2017; Wang et al., 2001).

## The impact of climate changes on African and Eurasian ecosystems

**Figure 3.**
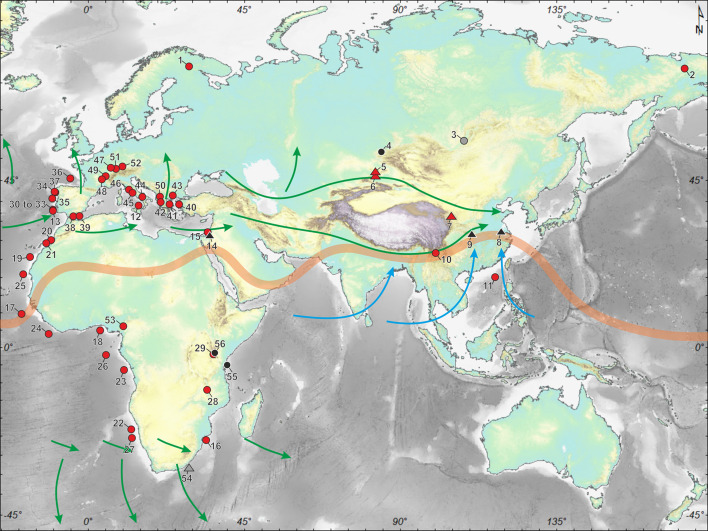
Map showing the locations of the palaeoenvironmental records discussed in the text and listed in Table 1 of the Supplementary Material. Red circles, pollen records; black circles, archaeological sites; grey circle, diatom record; red triangles, loess records; black triangles, speleothems. Orange line, Intertropical Convergence Zone (ITCZ). Green arrows, south and north westerlies. Blue arrows, Asian summer monsoon.

A wide array of regional palaeoclimatic records (Figure 3 and Table 1 of the Supplementary Material) show that global orbital and millennial scale climate changes were associated with shifts in the direction and intensity of the north and south westerlies that control the precipitation, and to a lesser extent temperature, from subtropical to high latitudes of both hemispheres (Blome et al., 2012; Li et al., 2019; Sánchez Goñi et al., 2016b; Urrego et al., 2015). These climate changes were also related to shifts in the position of the Intertropical Convergence Zone (ITCZ), leading to regional changes in monsoonal rainfall intensity in Africa and Asia (Caley et al., 2018; Cheng et al., 2016).

### Africa

Two chronologically well-constrained deep-sea pollen sequences, collected off the Congo and Limpopo Rivers, account for the long-term evolution of vegetation in western and eastern tropical Africa over the last 1 Myr at regional to subcontinental scale. The Congo fan record shows that during the Middle to Upper Pleistocene the vegetation in western equatorial Africa was increasingly influenced by the increasing amplitude of the glacial–interglacial variations (Dupont et al., 2001). The tropical forests surrounded by deciduous forests and woodlands were probably denser in the Middle Pleistocene compared with the latter period. These forests reduced during glacial periods, but remain in place over the last million years (Dupont, 2011; Dupont et al., 2001). A strong increase of lowland forest in the tropics occurred during the Last Interglacial MIS 5e (Dupont et al., 2001). The intermediate periods, such as MIS 5c and 5b, were marked by the abundance of mountain temperate forest (*Podocarpus;* Dupont, 2011) that contracted during the late Pleistocene glacials MIS 6 and MIS 4-2. Ericaceous scrubland developed during glacial maxima mainly resulting from low CO_2_ atmospheric concentrations (Jolly & Haxeltine, 1997).

**Figure 4.**
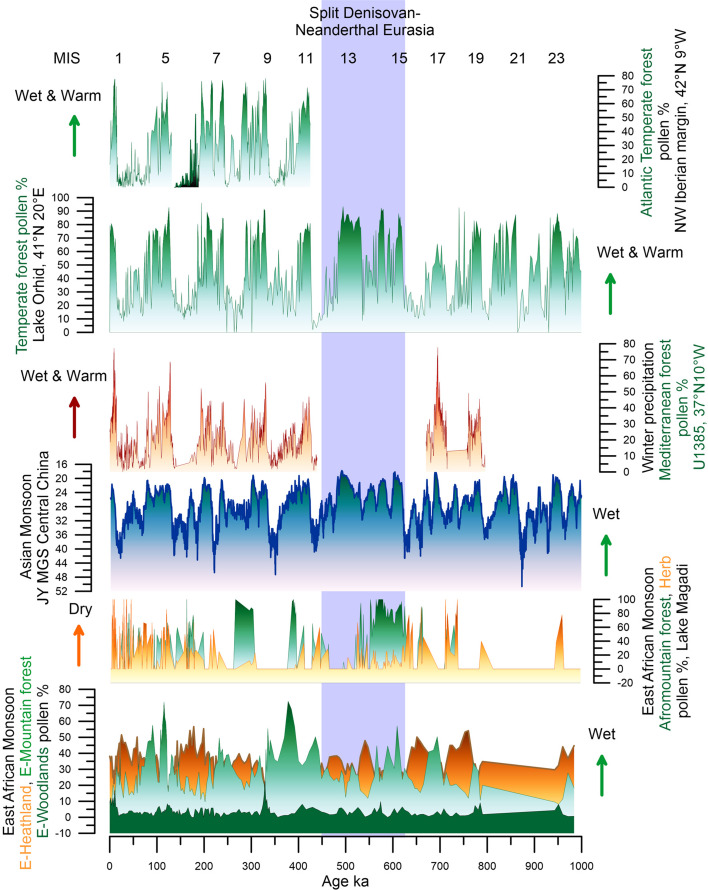
Palaeoenvironmental changes in Africa and Eurasia during the last 1 million years. (a) Deep-sea pollen record from core MD96-2048 (south-eastern African margin, 26°S, 34°E; Dupont et al., 2019). (b) Pollen record from Lake Magadi (Kenya, 2°S, 36°E; Owen et al., 2018). (c) Mean grain-size variations from the Jingyuan loess sequence (central China, 36°N, 105°E; Sun et al., 2006). (d) Mediterranean forest pollen record from the SW Iberian margin (composite record from sites U1385, MD01-2443 and |MD95-2042; Oliveira et al., 2017; Roucoux et al., 2006; Sánchez Goñi et al., 2008, 2016b, 2019). (e) Temperate forest pollen record from Lake Orhid (Albania; Wagner et al., 2019). (f) Atlantic temperate forest pollen record from the NW Iberian margin (Desprat et al., 2007, 2017).

The deep-sea pollen record off the Limpopo River shows that the mountain forest in the eastern tropical Africa was, as in its western part, abundant in southern Mozambique during the glacials of the last 800,000 years (Figure 4a). However, it was partially replaced by open ericaceous vegetation with some wet elements such as sedges (Dupont et al., 2019; Figure 4a). Different types of woodland in this region (coastal mangrove forest, riparian forest, Miombo dry forest and woodland) expanded during interglacials and their maximum expansion occurred after ~430 ka, and particularly during MISs 9e and 5e events (Dupont et al., 2019; Figure 4a).

To the north of the Limpopo basin, in the southernmost part of the East African Rift, the Lake Malawi pollen sequence shows nine phases of Miombo woodland and mountain forest alternating with savannah vegetation over the last 600,000 years related to hydroclimate change (Ivory et al., 2018). The *δ*^13^C_31_ record from the same sequence further indicates a progressively wetter climate over the past 1.3 Myr (Johnson et al., 2016). This was probably due to the progressive southward shifting of the ITCZ resulting from the long-term extensive glaciations towards the present. Further north, in the extratropical region of eastern Africa, the pollen record from Lake Magadi (Kenya) shows a decrease in the mountain forest at 575 ka indicating, contrasting with the Lake Malawi geochemical record, an increasing aridity punctuated by many drier and wetter cycles towards the Holocene (Owen et al., 2018; Figure 4b).

**Figure 5.**
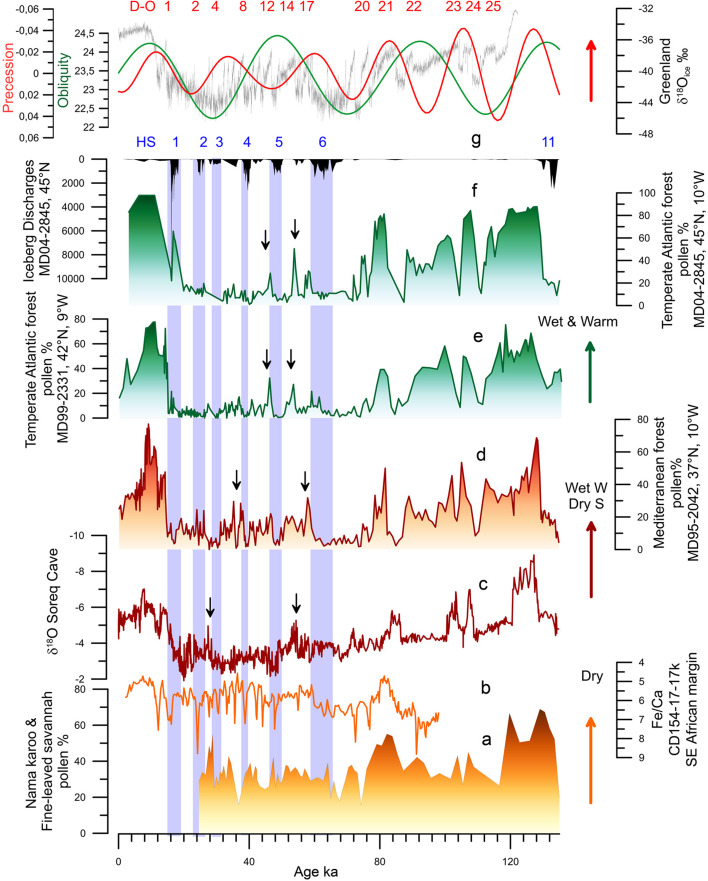
Millennial scale palaeoenvironmental changes in Africa and Eurasia during the last climatic cycle. (a) Deep-sea pollen record from core MD96-2098 (south-western African margin, 26°S, 13°W; Urrego et al., 2015). (b) Fe/Ca record from deep-sea core CD154-17-17K (south-eastern African margin, 32°S, 29°E; Ziegler et al., 2013). (c) *δ*^18^O from the Soreq cave speleothem sequence (Israel, 32°N, 35°E; Bar-Matthews et al., 1999). (d) Mediterranean forest pollen record from the SW Iberian margin (Sanchez Goñi et al., 2008). (e) Atlantic temperate forest pollen record from the NW Iberian margin (Sanchez Goñi et al., 2008). (f) Atlantic temperate forest pollen and ice-rafted debris records from the Bay of Biscay (western France; Sanchez Goñi et al., 2008 and unpublished data). (g) Precession index (red) and obliquity (green; Berger & Loutre, 1991) and Greenland ice core *δ*^18^O record (Rasmussen et al., 2014). Dansgaard–Oeschger (D–O) warming events (red) and Heinrich Stadial (HS) 11 and HSs 6–1.

For the last 450 kyr deep-sea pollen records distributed along the western and northern African coast show that the Saharan desert expanded during glacials and the grass and woodland savannah extended or shift to lower latitudes (Dupont, 2011). In the north, the Mediterranean forest area also expanded during interglacials. The intermediate periods, such as MIS 5c and 5b, were marked by the semi-desert expansion between the Sahara and northern Africa. For south-western Africa no long pollen records exist going beyond the last climatic cycle. The pollen content of a deep-sea core collected off the Orange River (Namibia) shows that the MISs 5e, 5c and 5a warming episodes were characterized by increases in aridity with the expansion of the nama-karoo and semi-desert towards the north and the east (Urrego et al., 2015; Figure 5a), while the MIS 5d and 5b cooling episodes and the following glacial period, MIS 4 and 3, were marked by pronounced increases of humidity and the subsequent development of the Fynbos.

Unfortunately, few high-temporal resolution pollen, and more generally palaeoclimatic records, exist accounting for the millennial to submillennial climatic variability in Africa throughout multiple glacial–interglacials cycles of the last 800,000 years. The most documented period is the last glacial represented by the pollen record of lake Bambili (Cameroon; Lézine et al., 2019) and a wax-based hydroclimate record from a deep-sea core collected off Port Elisabeth, in South Africa (Ziegler et al., 2013). The stability of the mountain forest at low altitude in western tropical Africa over MIS 4-2 is inferred from the Bambili record that also reveals the instability of the high altitude vegetation at that time (Lézine et al., 2019). The South African margin sequence shows an alternation between humid and dry periods in southern Africa probably in concert with cold GS and warm GI, respectively (Ziegler et al., 2013; Figure 5a).

In conclusion, this synthesis reveals that some African regions characterized by a present-day interglacial wet climate experienced drought periods, and viceversa. Furthermore, opposite hydroclimate changes existed at long timescales between the tropical south-eastern region (Mozambique) and the eastern extratropics (Kenya) as glaciations became stronger since 500 ka and the ITCZ progressively migrated southwards. This contrasting response is also observed, albeit more complex, during more recent and shorter periods of time (150–30 ka; Blome et al., 2012). The combined effect of the equator-ward displacement of the north and south westerlies and the southward displacement of the ITCZ during periods of ice volume increase, would have resulted in northern and southern Africa becoming wetter while tropical eastern and western Africa would become drier (Blome et al., 2012; Urrego et al., 2015; Figure 4a, 5a). However, in the core tropical and Sahara regions, forest and desert prevailed over the last one million years, respectively. These stable regions contrasted with those where substantial changes in vegetation occurred such as in northern, eastern and south-western Africa. Similar stable and unstable regions have been identified using a Global Climate Model Emulator that reconstructs the long-term evolution of climate with regional-scale dynamics at 1° resolution for the last 800,000 years (Krapp et al., 2019).

### Eurasia

**Figure 6.**
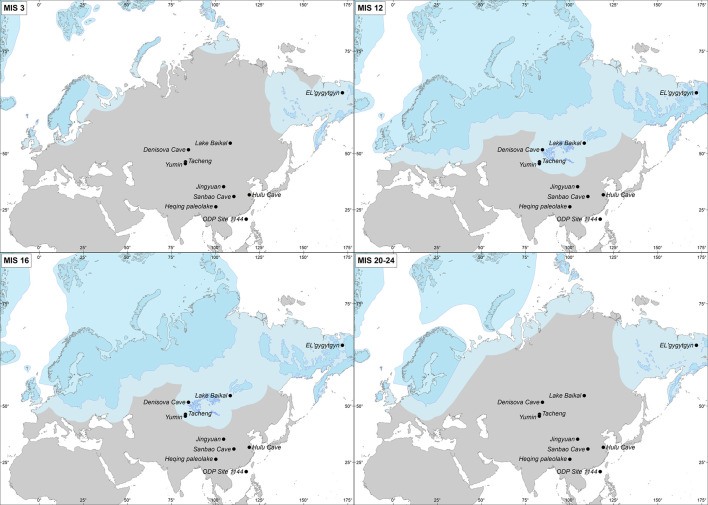
Reconstructed ice-sheet expansion (dark blue) during MIS 24–20 (928–790 ka, MIS 16 (650 ka), MIS 12 (450 ka) and MIS 3 (45 ka) from (Batchelor et al., 2019) and the associated extension of the permafrost (lighter blue) that is estimated at an average of 550 km extension towards the south based on the permafrost extension during the Last Glacial Maximum in Europe (Lindgren et al., 2016).

In contrast with the African continent, successive advancing and retreating Northern Hemisphere high latitudes ice-sheets are recorded in Eurasian geomorphological deposits and simulated by numerical modelling (Batchelor et al., 2019). Between 928–790 ka (MIS 20–24), most of the Eurasian continent was ice-free with ice-sheets concentrated in Scandinavia, northern Europe and the British Isles (Batchelor et al., 2019; Figure 6). Excluding the Scandinavian mountains that were an area of ice nucleation and subject to glaciations 8–10 times through the last million years, the Eurasian lowland landscapes (southern North Sea, south-west Russia, south-west Siberian Plain) were only covered by ice during the most extensive Eurasian ice-sheet advances, i.e. during MIS 16 (~650 ka, almost down to the Black Sea), MIS 12 (~450 ka, down to south Moscow), MIS 6 (~150 ka, down to south Kiev) and the Last Glacial Maximum (20 ka), and to a lesser extent of European Ice Sheets (EIS) during MIS 10 (350 ka) and MIS 8 (250 ka; Hughes et al., 2020). The Eurasian ice-sheets reached their most southerly position in western Siberia at ~650 and 450 ka (Batchelor et al., 2019; Figure 6). This large ice extension was probably the result of the relatively high amount of winter rainfall during the period MIS 17–11, detected in the study of north-western and central China Loess Plateau sequences, feeding the ice caps (Sun et al., 2006; Figure 4c). Further north-east, the study of littoral benthic diatom assemblages and lithogenic sediments from the Lake Baikal sedimentary sequence reveals, in contrast, a virtually continuous interglacial climate in central eastern Siberia between MIS 15a and 11 (~580–380 ka), with an apparent lack of extensive mountain glaciations (Prokopenko et al., 2002). This result was unexpected because global climatic data for the MBE, between MIS 12 and 11 (480–380 ka), indicate a substantial change between an extremely cold glacial period and an unusually warm interglacial period. The interglacial conditions in this region are nevertheless supported by recent research demonstrating that continuous permafrost only developed after 400 ka when the Arctic sea ice was present (Vaks et al., 2020). This was certainly associated with the increasing amplitude of glacial–interglacial cycles during the MBE.

During the last glacial inception (MIS 5d, 115 ka) glaciers and ice-sheets developed in continental interiors (i.e. NE Asia and eastern Europe) whilst large ice-sheets close to Atlantic moisture sources, i.e. the southern margin of the Laurentide Ice Sheet and the western EIS, reached their maximum extent at the Last Glacial Maximum (23–19 ka) or early in the last glacial cycle (Batchelor et al., 2019; Hughes et al., 2013). Ice-sheets in eastern Europe and NE Asia were probably of similar size or even more extensive in MIS 5b and 5d (115–90 ka) compared with MIS 4 (73–60 ka). This spatial pattern suggests that Late Pleistocene glaciation may have been initiated in the north Pacific region before spreading to the North Atlantic region (Batchelor et al., 2019), but it also suggests that Europe was more glaciated during the last glacial period than any time over the last 1 Myr. Also, the partial marine-based nature of the EIS seems to be marked by a higher susceptibility to rapid and unstable ice-sheet collapse (Batchelor et al., 2019) supporting the evidence that climate was particularly variable during the last glacial period, and particularly during its middle part (MIS 3; Bond et al., 1999; Figure 6).

The impact of the cyclic ice-sheet advances and retreats on Eurasian ecosystems is documented by a number of long pollen records extending beyond the last climatic cycle. However, most of these sequences are located south of 47°N, and only records the evolution of the southern European vegetation. Three long pollen records are available for tracing the vegetation of continental Asia: one from south-eastern Asia (Heqing Basin), another, albeit fragmentary, from north-eastern Siberia (El'gygygytn), and a third one collected in the South China Sea. Similar to the tropical African regions, the pollen analysis of the Heqing Basin sequence (2190 m asl), covering the last million years (Xiao et al., 2007), shows that in tropical Asia the present-day altitudinal vegetation belts, dominated by the montane conifer and broadleaved mixed forests and shrub-grassland savanna in the warm-dry valleys, shifted but persisted despite the glacial–interglacial cycles affecting the East Asian and Indian summer monsoons (Cheng et al., 2016; Zhisheng et al., 2011). Further south, close to Taiwan the deep-sea pollen record from the South China Sea (Sun et al., 2003) indicates that the climate between 800 and 400 ka was, as in tropical Africa, more suitable for growth of forests with tropical nature than after 400 ka, but no distinct glacial–interglacial variations are recorded in the tropical montane and boreal conifer forests either. The sudden expansion of evergreen and deciduous Fagaceae trees (mainly *Quercus* and *Castanopsis*) about 355 ka, might imply strengthened seasonality and a cooler climate than previously. The relative climatic cooling during this later period is also confirmed by the increase of the boreal conifer trees. In glacial times evergreen broadleaved forests were probably more open than today but still survived in the southern coastal areas of China, and the emerged part of the continental shelf was mainly covered by grassland (Sun et al., 2003). Much further north, the fragmentary pollen record from Lake El'gygygtyn shows in contrast that MIS 11c interglacial (~400 ka) was marked by the strong development of spruce indicating a particularly warm and wet super-interglacial in north-eastern Siberia (Melles et al., 2012).

Long European pollen sequences and deep-sea pollen records from its margin (de Beaulieu et al., 2001; Helmens, 2014; Sanchez Goñi et al., 2018; Tzedakis et al., 2006; Wagner et al., 2019) show an alternation between glacial periods, dominated by semi-desert in southern Europe, below 40°N, and steppe-tundra/heathlands to the north, and interglacial periods when Mediterranean and temperate /boreal forests expanded in southern and northern Europe, respectively (Figure 4d–f). In southern Iberia the magnitude of the Mediterranean forest development substantially differed from one interglacial to another (Figure 4d), while in north-west Iberia the magnitude of the Atlantic temperate forest expansion was similar (Figure 4f; Desprat et al., 2017). Excluding the Lake Orhid pollen record (Sadori et al., 2016; Wagner et al., 2019; Figure 4e), located in a present-day ecotone between Mediterranean and continental climate, these results coincide with what is observed in the European sequences of Tenaghi Philippon and Praclaux located above 40°N (de Beaulieu et al., 2001; Reille, de Beaulieu, Svobodova, Andrieu-Ponel, & Goeury, 2000; Tzedakis et al., 2006). They show similar expansions of the temperate forest during the interglacials of the last 400,000 years. In the Mediterranean region below 40°N, the Holocene (MIS 1, ~10 ka), the last interglacial (MIS 5e, ~128 ka) and MIS 17c (~690 ka) have been the most forested/wettest periods followed by MIS 9e (~335 ka) and MIS 7e (~240 ka). MIS 11c (~420 ka) and MIS 19c (~784 ka), marked by a limited expansion of the Mediterranean forest would have been the driest (Oliveira et al., 2017, 2018; Roucoux et al., 2006; Sánchez Goñi et al., 2016a, 2019). These data suggest that temperatures were similarly warm in the European mid-latitudes during the interglacials of the last one million years, whereas the amount of winter precipitation, and therefore of forest cover in the south, was quite variable. Interestingly, both Mediterranean and Atlantic/temperate forests lasted much longer, almost 30,000 years, during MIS 11c than during the previous and subsequent interglacials marked by an average duration of 10,000–15,000 years.

Most of the Eurasian millennial-scale climatic records derive from Europe and its margins (e.g. Margari et al., 2010; Oliveira et al., 2017; Sanchez Goñi et al., 2008, 2016b). For Asia, the best documented millennial scale climatic variability is provided by speleothems (Cheng et al., 2016; Wang et al., 2008) and loess sequences (Li et al., 2019), no pollen sequence has been published thus far (Sanchez Goñi et al., 2017). The Asian records show respectively alternating increasing and decreasing precipitation, as the result of stronger and weaker influence of summer monsoon in the south (Cheng et al., 2016) and the westerlies in the north (Li et al., 2019), concomitant with warming and cooling D–O events in the Greenland and North Atlantic European regions including the eastern Mediterranean (Bar-Matthews et al., 1999; Figure 5b). Deep-sea pollen records from the western European margin and the Mediterranean Sea directly compared with ice and ocean climatic indicators have unequivocally shown that suborbital cooling events in the North Atlantic and Greenland corresponded in western Europe with herbaceous community expansion, and warming events with forest development (Combourieu-Nebout et al., 2002; Roucoux, 2000; Sánchez Goñi et al., 2000, 2002, 2008; Figure 5c–e).They also demonstrate that vegetation responded rapidly, within 100 years of the D–O cycles and HEs, and that there was a dynamical equilibrium between vegetation response and climate change for short periods of forcing. Notably, below 40°N, Mediterranean forest, mainly deciduous and evergreen *Quercus*, reached its maximum development at the onset of the Last Interglacial (MIS 5e), the D–O 24, D–O 21, D–O 17–16, D–O 8–7 and D–O 1 warming events, and the onset of the Holocene (MIS 1), indicating the occurrence of enhanced hot-dry summers and wet-cool winters at that times (Figure 5c). The Atlantic sites, above 40°N, showed a contrasting pattern: at D–O 12 and D–O 14 the Atlantic forest, mainly *Betula*, deciduous *Quercus* and *Pinus*, experienced a strong development while the impact of D–O 16–17 and D–O 7–8 warming was rather limited (Figure 5d–e). The compilation of these Mediterranean and Atlantic deep-sea pollen records with European pollen sequences (Duprat-Oualid et al., 2017; Fletcher et al., 2010) reveals, despite the independent and sometimes uncertain chronologies of individual terrestrial records, that there was a spatial variability in the amplitude of the forest expansions for any given D–O warming of the last glacial period (Fletcher et al., 2010; Sanchez Goñi et al., 2008). The maximum Mediterranean forest expansions occurred during GI 17–16 and GI 8 synchronous with low precession values that promote marked seasonality. Thus, there was an optimal forest development associated with a strong richness in sclerophyllous plants such as evergreen *Quercus*, *Olea*, *Cistus*, *Phillyrea*, *Coriaria myrtifolia* and *Pistacia* (Fletcher & Sanchez Goñi, 2008). GIs occurring during precession maxima were marked in turn by the abundance of the less drought-tolerant Ericaceae (heather) typical of a weak seasonal climate (Fletcher & Sanchez Goñi, 2008). In contrast, maximum expansion of the Atlantic forest, detected during GI 12 and GI 14, was synchronous with maxima in obliquity that promotes increase of annual temperatures. During the GSs, the western Mediterranean region was characterized by semi-desert plants (mainly *Artemisia*, Chenopodiaceae and *Ephedra*) while simultaneously, Ericaceae and Poaceae dominated the vegetation of north-western Iberia. The composition of the pinewoods of south-western/central Iberia during the last glacial also varied in response to abrupt climate changes (Desprat et al., 2015). *Pinus nigra* was dominant throughout the last glacial period but *P. sylvestris* was more abundant during the GSs and HSs, and Mediterranean pines (*P. pinaster*, *P. pinea*, *P. halepensis*) during the GIs and early to mid-Holocene. Further north and east, *Artemisia*, Cyperaceae and *Calluna* dominated in Western Europe during the cold periods, whereas *Betula*, deciduous *Quercus* and conifers (*Pinus*, *Abies*, *Picea*) expanded slightly in the landscape during warming episodes (Fletcher et al., 2010). In southern Germany, recent pollen evidence shows the expansion at low altitudes of *Betula*, deciduous *Quercus*, Cupressaceae and *Pinus* during GIs (Duprat-Oualid et al., 2017). Ice-free regions of Fennoscandia were marked by a *Betula*-dominated vegetation, including tree birch (Helmens, 2014). These sequences illustrate the spatial floristic diversity of Europe in response to the D–O cycles. Interestingly, the latitudinal boundary between the Atlantic and the Mediterranean vegetation during the last glacial period seems to have been similar to that at present day, i.e. 40°N.

In conclusion, the combination of the aforementioned evidence, in agreement with recent projections (Krapp et al., 2019), highlights the strong instability of most of the Eurasian ecosystems at orbital and millennial time scales marked by an alternation of afforestation and herb development concomitant with substantial ice-sheet contraction and expansion, respectively. The most important and southernmost Eurasian ice-barriers reached south-western Russia and occurred during MISs 16, 12 and 6, but the central Siberian region was ice-free up to 400 ka. In contrast, the core tropical Asian regions and those sparsely vegetated such as southern Eurasian deserts and a few fragmented core boreal forest habitats have been among the most stable regions in the world. The pollen record of forest cover of the different European interglacials above 40°N was similar, whilst in the Mediterranean region south of 40°N, the maximum development of the forest occurred during MISs 17, 5 and 1. Millennial-scale changes in forest cover were more frequent and larger during the last glacial period (MIS 5d-2) than during MIS 6 (Margari et al., 2010) probably as a consequence of the instability of the EIS. A contrasted forest response to the rapid warming events of the last glacial is observed in Europe.

## A causal link between climate changes and biological and cultural human evolution?

So far research has often been limited to proposing a causal link based on the presumed contemporaneity between a climatic event or series of events and biological (extinction or emergence of new species, biogeographic distribution) or cultural phenomena (appearance or decline of technical systems, modes of social organization, changes in the geographical distribution of these features). If the contemporaneity between a climatic and a cultural/biological event does not constitute *per se* a proof that the former is the cause of the latter, a synchronism between environmental and archeological changes is necessary *a priori* to test the climatic hypothesis. However, inferring causal links between, on the one hand, environmental and biological/cultural changes, and, on the other, environmental changes and migration waves is hampered by the uncertainties of the different numerical and radiometric dating methods applied to palaeoclimatic and archaeological records and the debated molecular clock in genetics based on constant rate of mutations. Moreover, the paucity and low resolution of most palaeoenvironmental and archaeological evidence still preclude firm conclusions on the processes underlying adaptation, biological and cultural evolution and exit routes of the genus *Homo* since the last million years (e.g. d’Errico et al., 2020). In recent years, different modelling approaches have been developed to help us in deciphering the possible climatic processes underlying biological and cultural human evolution and the dispersal of *Homo* out of Africa. In the following subsections, the possible impact of climate changes on four key events in human evolution are addressed.

### The multiregional emergence of H. sapiens and its dispersal out of Africa

**Figure 7.**
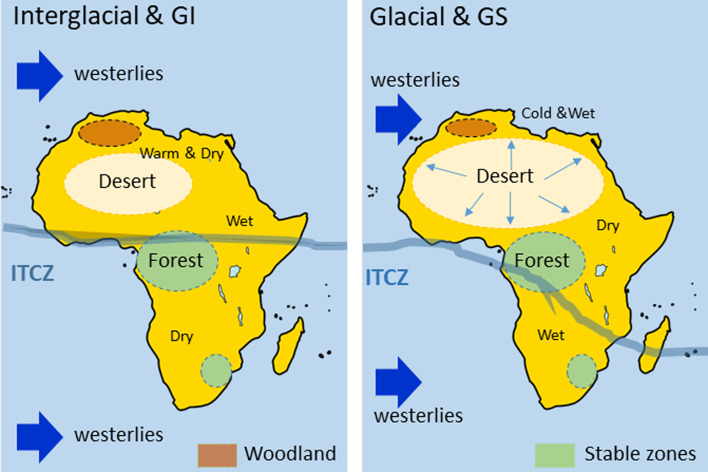
Schematic representation of the environmental changes in Africa at orbital and millennial-type scales showing the contrasting regional response. ITCZ: Intertropical Convergence Zone.

Data and projections presented above show that unstable environments were located in northern and southern Africa as well as in extratropical eastern Africa (Figure 7). These regions witnessed the first morphological traits characterizing *H. sapiens* since the origin of his first lineage at least 500 ka, and genetic data confirm these findings (Scerri et al., 2018). The emergence of early Middle Stone Age (MSA) cultures, dated at ~350 ka in the Olorgesailie basin (Kenya), coincides with enhanced wet-dry variability and a progressive increase in aridity since ~600 ka as well as a markedly different faunal community (large mammals turnover) compared with that of Acheulean occupations (Owen et al., 2018; Potts et al., 2018). Climate change seems also to play an important role for the replacement of the Still Bay technocomplex with Howiesons Poort MSA industries that developed in southern Africa from 76 ka to 59 ka, encompassing the MIS 5a/4 and MIS 4/3 transitions. Based on the aforementioned palaeoclimatic evidence it is known that South Africa was drier during interglacials and wetter during glacials as indicated by the development of the semi-desert and the fynbos, respectively (Urrego et al., 2015; Figure 7). The Still Bay industries thus developed during a glacial, moist climate (76–71 ka) and the Howiesons Poort during the subsequent drying period marked by the expansion of the semi-desert between ~66 and 59 ka (d’Errico et al., 2017). Using an eco-cultural niche modelling approach, a clear niche expansion of the Howiesons Poort culture is projected at ~65 ka. This culture is marked by less specialized tools than those of the Still Bay, emphasizing the flexibility of the Howiesons Poort craftspeople to produce innovations that allow a population to shift its ecological niche (d’Errico et al., 2017). These new data contrast with earlier hypotheses that suggest that both Still Bay and Howiesons Poort industries appear during arid (e.g. Ambrose & Lorenz, 1990; McCall, 2007) or wet (Chase, 2010) conditions previously inferred for MIS 4 in southern Africa. Thus, unstable environments with repeated habitat fragmentation, isolation and admixture seems to be optimal places for cultural and biological evolution of *Homo* leading to the multiregional origin of *H. sapiens* (Scerri et al., 2018).

However, an alternative hypothesis explaining the development of new industries have been recently proposed (Roberts et al., 2020). The heterogeneous environments of the tropical coastal Africa inferred from the combination of zooarcheological studies with isotopic stable analyses of human and faunal tooth enamel of the Panga ya Saidi site (Kenya) may have represented an important refugium for populations during the increasing climatic variability of the Late Pleistocene. This recent study proposes that the transition from MSA to Late Stone Age technological industries was not related to a change in the environmental resources. Both populations consistently utilized tropical forest and grassland biomes, further suggesting that not only grassland and marine resources (Marean et al., 2007) were key drivers behind the material culture innovations and the rapid expansion of *H. sapiens* within and beyond Africa (Roberts et al., 2020).

While heterogeneous and relatively stable tropical regions would have facilitated the early waves of human migration towards the Levant and Arabia as early as 177 ka (Hershkovitz et al., 2018) and towards Eurasia at ~120 ka (Liu et al., 2015), other studies (Tierney et al., 2017) claim that the ‘out of Africa’ migration could have been facilitated by warm and wet conditions from 120 to 90 ka. This hypothesis is supported by fossil and lithic evidence, contrasting with genetic studies, which constrain the primary out-of-Africa event during a purported cold and dry time (~65–55 ka). Data show however that the putative 65–55 ka cold and dry time period was marked by millennial to submillennial scale climatic changes superimposed to a period marked by precession minima. This period encompasses two distinct main phases: the very cold and dry HS 6 (64–60 ka) in the North Atlantic borderlands and the warmer and wetter GIs 17 and 16 (~59–56 ka) when the Near East (Levant) and the Mediterranean region experienced an amplified increase in winter precipitation and forest development at the time of minima in precession (Bar-Matthews et al., 1999; Fletcher et al., 2010; Langgut et al., 2011; Sanchez Goñi et al., 2008; Figure 5b). This wet period may also have facilitated the out of Africa movement at that time as suggested by genetic data even if it is not currently supported by lithic and fossil evidence. Interestingly, a recent numerical human dispersal model, which is forced by spatiotemporal estimates of climate and sea-level changes over the past 125 kyr, reveals that repeated orbitally driven wetting of northern Africa around 106–94, 89–73, 59–47 and 45–29 ka favoured the development of vegetated corridors and therefore the recurrent exit of *H. sapiens* towards Eurasia (Timmermann & Friedrich, 2016). Contrasting with this hypothesis, a comparison between the demographic structure of African hominins and the environmental changes between 140 and 30 ka indicates that during arid intervals between 120 and 80 ka, relative site abundances in tropical Africa increase, perhaps tracking the local effects of forest fragmentation and grassland expansion, whereas those of eastern and northern Africa decrease (Blome et al., 2012). Processes of forest fragmentation may explain population isolation, but also contact between populations and human dispersal as the result of corridor formation as suggested by regional genetic data. Both the drying- and wetting-driven development of corridors indicate a complex intertwining of climate and human population movement between the African regions and out of Africa.

### The emergence of Neanderthals

Neanderthals and Denisovans emerged in Eurasia from a common ancestor before 400 ka until they were replaced by *H. sapiens* after 40 ka (Slon et al., 2018). DNA sequences of Neanderthals and the Denisova individual found in the Denisova cave, located in central Siberia not far from Lake Baikal, diverged on average 640,000 years ago (Reich et al., 2010), and it has been proposed that the origin of Neanderthals at ~600–400 ka, carrying the Acheulean technology into Europe, could have emerged as a result of the enhancement of both glacial and interglacial phases recorded at that time (Hublin, 2009). Geological archives and model simulations presented above show that at this time Eurasian palaeoenvironments were marked by the southernmost extension of the ice-sheets, with permafrost probably developing as far south as the Black Sea (Figure 6). This ice-sheet configuration may have produced a north–south ice barrier and the consequent genetic drift isolating in two groups, Neanderthals in the west and Denisovan in the east, the population of *H. ergaster* that may have migrated from Africa at 1.7 Ma and lived in an ice-free Eurasia until ~600 ka (Figure 6). More recent genetic data suggest alternative splitting times (Prüfer et al., 2017) that can also be explained by environmental changes. The split time between the Vindija Neanderthal and *H. sapiens* is estimated at between 520 and 630 ka and, therefore, during the first ice-barrier formation dated to ~650 ka (Figure 6, MIS 16), while the second north–south ice barrier at ~450 ka (Figure 6, MIS 12) would trigger the split between the Vindija Neandertal and the Denisovans estimated at 390–440 ka, both in agreement with previous estimates using the Altai Neandertal (Prüfer et al., 2017). Denisovans would have remained in eastern Eurasia and migrated towards the south-east where stable environments with south-eastern subtropical and tropical forest persisted under the orbital-scale control of the summer monsoon (Sun et al., 2003). Following the genetic drift Neanderthals would evolve in Europe where anthropological features and encephalization are clearly established after the MIS 12 glaciation (Hublin, 2009) perhaps as the result of their adaptation to the numerous and large amplitude of climatic changes recorded since then. Archaeological records demonstrate more occupations, evidence of new subsistence behaviours and technical innovations (core technologies, increase in light-duty tools) during interglacial MIS 11, at around 400 ka, associated with an early regionalization of traditions (Moncel et al., 2018) that may have been the result of an exceptionally long interglacial following the severe MIS 12 glacial period. The MIS 11 warm ‘super-interglacial’ in the polar regions was not, however, exceptionally forested in southern Europe as winter precipitation was relatively low (Oliveira et al., 2018; Wagner et al., 2019). Grasslands expansion in this region could have provided high biomass availability and therefore large herbivore population with the consequent increase in the demography of Neanderthals and cultural diversity. High availability of resources and population increase have been put forward as mechanisms triggering cultural diversity (Collard & Foley, 2002) and innovations (Richerson et al., 2005), respectively.

### The replacement of Neanderthals by H. sapiens

After surviving several hundred thousand years of long-term and rapid environmental changes in Europe and the Near East, Neanderthals disappeared at ~36 ka. Despite a lively debate on the causes of their disappearance, such as abrupt climate change, volcanic eruption or epidemics (Finlayson et al., 2006; Lowe et al., 2012), the latest works suggest that *H. sapiens* carrying the Proto-Aurignacian culture gradually replaced Neanderthal populations carrying the Mousterian, Châtelperronian and Uluzzian technologies during the GI 10 (42 ka) and the subsequent GS 10 (40 ka; d’Errico & Banks, 2015). The Lower Aurignacian superseded the Proto-Aurignacian in the following cold period, the HS 4, 40–38 ka. Neanderthals survived several millennia up to the end of GI 8 (~36 ka) in southern Iberia, a region that was not colonized by *H. sapiens* until that time. In this region, the scarcity of Mousterian sites dated to HS 4 and their reappearance at the end of this period suggest a contraction of the Neanderthal population followed by a momentary expansion, before its definitive disappearance perhaps 36 ka.

Based on the comparison between the archaeological evidence from southern Iberia and deep-sea pollen records from its margin, d'Errico & Sanchez Goñi (2003) proposed an ecological hypothesis to explain the late survival of Neanderthals in southern Iberia: the low plant and animal biomass characterizing the semi-desert landscape of HS 4 was an obstacle to the penetration in southern Iberia of *H. sapiens*, an obstacle that would have separated them from the Neanderthals who lived in this region for more than a millennium. Characterized by a very low biomass, the environments of southern Iberia during HS 4 (40–38 ka) could only feed a small number of mammals and support low populations of hunter–gatherers. On the contrary, the contemporary steppes of northern Iberia and especially those of western France were rich in grasses and maintained large herds of herbivores (bison, horses, reindeer). With a mode of subsistence based on hunting large steppe mammals, the Aurignacians colonizing the north of Iberia probably saw no advantage in moving towards an arid south poor in natural resources. Neanderthals responded to the aridification of their regions by reducing their numbers and confining themselves to refuge areas that allowed them to maintain a way of life based largely on the systematic exploitation of local resources, and creating a barrier between modern humans in the north and the Neanderthals in the south. The following GI 8 warmer and wetter phase, would have allowed contact, and thus competition, between the two populations and resulted, as in the rest of Europe, in the disappearance of the Neanderthals.

**Figure 8.**
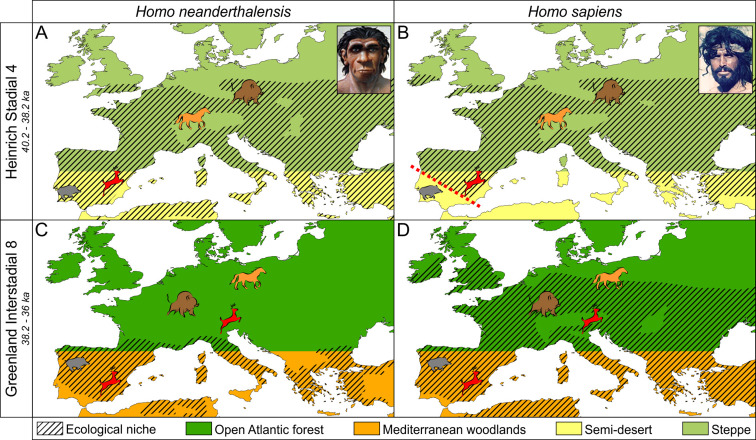
Representation of the simulated expansion and contraction of the eco-cultural niches of Neanderthals and *Homo sapiens* in western Europe and, particularly in the Iberian Peninsula during HS 4 and GI 8 (after Banks et al., 2008). *H. sapiens* did not reach southern Iberia because of the low animal biomass during HS 4 allowing Neanderthals several years of survival in this region. With the afforestation of the GI 8, *H. sapiens* colonized southern Iberia and was in competition for the same ecological niches occupied by Neanderthals leading to their niche contraction.

The ecological barrier during HS 4 disappeared with the beginning of the GI 8 temperate phase, which contradicts the idea of a slow degradation of the climate and its possible role in the disappearance of the Neanderthals, as proposed by some researchers (Finlayson et al., 2006). The hypothesis of competition between these two human groups therefore remains the most plausible, but the possible reasons for the success of *H. sapiens* (higher rate of reproduction, more advanced technology, greater aggressiveness, etc.) remain speculative. The hypothesis of competition has been corroborated by an eco-cultural niche modelling approach (Banks et al., 2008; Figure 8), and more recently by a numerical model of interspecific competition including the ‘culture level’ of a species as a variable that interacts with population size. This model shows that relative culture level, rather than epidemics or climate change, could have caused the eventual exclusion of Neanderthals by modern humans (Gilpin et al., 2016). In agreement with previous models, a new spatially resolved numerical hominin dispersal model with empirically constrained key parameters that simulates the migration and interaction of *H. sapiens* and Neanderthals during the rapid D–O events shows that these climatic events were not the major cause of the disappearance of Neanderthals. Further, a series of parameter sensitivity experiments conducted with this model shows that a realistic disappearance of Neanderthals requires to choose *H. sapiens* as a more effective population in exploiting scarce glacial food resources as compared with Neanderthals (Timmermann, 2020).

The disappearance of the Neanderthals therefore does not appear to have been caused by climate change. Paradoxically, it is even a climatic deterioration which, by interposing inhospitable environments between the two populations in Iberia, would have protected the Neanderthals from competition with *H. sapiens* by giving them a few millennia of relief and promoting their survival in a dead-end situation. Climate change does not seem to have been the determining factor in the colonization of Europe by *H. sapiens* either, since this process took place over several millennial climatic cycles. At a regional scale, however, climate could certainly have conditioned the replacement, as it was probably the case in the Mediterranean region. This conclusion is logical if it is remembered that before the arrival of *H. sapiens*, the Neanderthals were repeatedly subjected to similar strong climatic changes and experienced cold periods much longer than the HS 4.

### New technical strategies during the Upper Palaeolithic

The last ice age witnessed the arrival in Europe of *H. sapiens* at ~42 ka and, subsequently, the development of the so-called Upper Palaeolithic cultures (d’Errico & Banks, 2015). Testing the hypothesis that climate changes could have had an impact on the population demography and the emergence of new cultures, requires the comparison between the distribution of the number of European Palaeolithic sites dated between 8,000 and 36,500 ^14^C AMS years BP, and different palaeoclimatic records.

**Figure 9.**
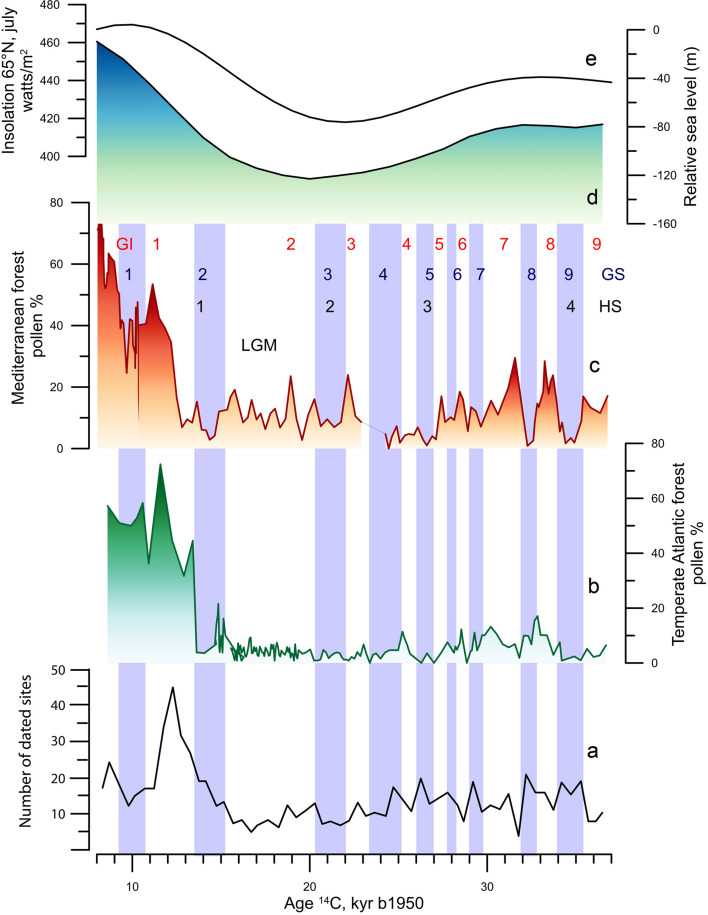
Comparison between palaeoenvironmental changes in western Europe and the number of dated archaeological sites by 500 year intervals during the last 37,000 ^14^C years. Due to the uncertain age reservoir of foraminifera-based ^14^C marine samples, we compare archaeological and marine palaeoclimatic records plotted against raw ^14^C ages. (a) Number of dated archaeological sites (d’Errico et al., 2006). (b) Mediterranean forest pollen record from the SW Iberian margin (Sanchez Goñi et al., 2008). (c) Atlantic temperate forest pollen record from the NW Iberian margin (Sanchez Goñi et al., 2008). (d) Relative sea-level changes as an indicator for ice-volume changes (Waelbroeck et al., 2002). (e) changes in insolation in July at 65°N (Berger & Loutre, 1991). LGM: Last Glacial Maximum

The comparison of the temporal distribution of sites, at 500 year intervals, with the benthic foraminiferal δ^18^O record shows long-term, orbital, close general patterns (Figure 9a, d, e). The increase in ice volume is concomitant with the decrease in the number of sites indicating a population contraction due to the reduced extent of areas with resources exploitable by Palaeolithic hunters (d’Errico et al., 2006). This hypothesis is confirmed by taking into account the latitudes and altitudes of the archaeological sites (d’Errico et al., 2006). The reduction in the number of sites during the Last Glacial Maximum corresponds to the compression towards lower latitudes and altitudes of their distribution. This compression clearly reflects the effect on the biosphere of increasing ice volume. The population reduction during the Last Glacial Maximum is certainly overestimated because we lack information on the colonization of large areas of land as a result of sea-level lowering. Located at low altitude and often consisting of plains, these areas could support steppe vegetation suitable for certain species of ungulates and their predation by Palaeolithic populations.

At the millennial timescale, the comparison of the number of European Upper Palaeolithic sites with the GI and GS phases dated by ^14^C AMS in the deep-sea pollen records MD95-2042 (Shackleton et al., 2004) and MD99-2331 (Naughton et al., 2009; Figure 9a–c) shows that the population density decreased during warming events and contemporaneous forest development. Each afforestation reduced the biomass of ungulates (d’Errico et al., 2006), the main resource of Paleolithic hunters (Bocherens et al., 2005; Richards et al., 2000, 2001). By reducing the forest cover and leaving steppes to develop, the periods of cooling and drying were characterized in the mid-latitudes by an increase in ungulate biomass, causing of a human demographic boom. This interpretation is supported by present-day estimates of ungulate biomass and human population density of historical hunter–gatherers with temperate steppes allowing for a higher ungulate biomass, which results in a higher human population density (d’Errico et al., 2006).

The increase in the number of sites seen in the early part of the Bølling–Allerød interstadial warm phase (GI 1, 14.7–12.8 ^14^C ka) is undoubtedly related to the recolonization of high latitudes and altitudes at the time of the retreat of glacial and periglacial zones (Figure 9). However, as soon as a real forest colonization is established, at around 12,000 14C AMS years BP, a further reduction in the number of sites is seen, brought about by a reduction in the ungulate biomass. Momentarily halted by the deforestation produced by the cold episode of the Younger Dryas Stadial (GS 1, 12.8–11.7 ^14^C ka), this tendency to reduce the number of sites seems to be repeated as soon as warming resumes, in the early Holocene. The increase in the number of sites in the early post-glacial period, in a very forested environment, can be related to new modes of adaptation, such as intensive harvesting that exploit the entire terrestrial and marine resources and makes the demography of human groups independent of ungulate biomass. These results further show that GS marked by a drop in temperature and rainfall, considered of high ecological risk for historical hunter–gathers populations (Cashdan, 2001; Collard & Foley, 2002; Mace & Pagel, 1995; Nettle, 1998), should stimulate the maintenance of the same cultural and linguistic features over larger areas while GI warm periods are expected to lead fragmentation of these ethno-linguistic units. The analysis of the distribution of body ornaments in Europe will allow in the near future to test this scenario.

In conclusion, the long-term variation in the number of Upper Palaeolithic sites would reflect the population density, and that the high population density depends both on the ice-free surface area offering hunting resources and, on a finer timescale, the development of steppes during the cold phases of the D–O cycles offering a higher ungulate biomass. Richerson et al. (2005) have hypothesized that during GI, the increase of forest produced more resources allowing the increase in populations and therefore in innovations. While in South Africa this scenario seems to be the case (Ziegler et al., 2013), innovations in Europe were produced rather during cold and dry periods. A preliminary comparison between environmental and cultural evidence has shown a certain synchronicity between the development of each new ‘culture’ of the Upper Palaeolithic and the beginning of a new HS. A new robust chronological framework, based on Bayesian methods and a critical evaluation of dated archaeological context, proposes that the succession of archaeological cultures between 32 and 21 ka cannot be robustly associated with millennial-to-centennial climate changes (Banks et al., 2019). However, the pertinence of the cultural units considered by Banks and colleagues to test the climatic hypothesis is debatable. These units correspond in some cases to the whole chronological extent of a cultural unit or technocomplex identified by French archaeologists (e.g. Badegulian), in other cases to subcultural units (e.g. Upper Solutrean) determined on the basis of dated changes in the cultural evolution of one of these technocomplexes. Since the degree of cultural continuity/discontinuity between these different entities is only qualitatively established and does not formally take into account changes in each adaptive domain it is problematic to discard the influence of climate as a triggering or co-triggering factor.

## Conclusions

This review, albeit not exhaustive and suffering from the paucity of high-resolution pollen records from Africa and Asia, has highlighted the following features:

1. A contrasted regional response of African environments to long-term and millennial-scale climate changes of the last million years. Glacial and interglacial, and probably stadial and interstadial, periods respectively triggered afforestation and semi-desert expansion in South Africa and southern Mozambique. In the north, west and eastern African regions climate changes led to an opposite vegetation response: glacial and interglacial, and probably stadial and interstadial, periods were respectively contemporaneous with the expansion of grasslands and woodland development. Both unstable environments in southern and northern Africa and stable but heterogenous landscapes in the eastern tropical region may have provide the optimal conditions to trigger biological and cultural innovations underlying the multiregional origin of *H. sapiens*. During interglacials the development of both vegetated corridors in north and eastern Africa and the Levant and forest fragmentation in southern Africa may have promoted population admixture and facilitated the ‘out of Africa’ of *H. sapiens*.
2. In contrast to the African continent, Eurasia was affected by strong ice advances and retreats over the last one million years. An alternation between forested and steppe periods dominated interglacial and glacial landscapes, respectively. North of 40°N, interglacials were similarly forested, while in the Mediterranean region below 40°N the forest cover varied. The large glaciations at 650 ka and 450 ka and the associated permafrost development may have produced a north–south ice-barrier in western Eurasia as far south as the Black Sea that could promote a genetic drift in the populations living in Eurasia and therefore the split between Neanderthals and Denisovans at that time as shown by genetic data. One way to test this hypothesis would be to check whether the same genetic drift is also recognize in any other animal and plant taxa.
3. Superimposed on the glacial–interglacial cycles, millennial-scale variability has been a pervasive feature during the last one million years, but few long regional palaeoenvironmental records exist for this interval. The best documented millennial-scale variability is that punctuating the last glacial period, first recorded in Greenland and the North Atlantic, and the best documented region is Europe. During GSs the expansion of semi-desert and steppe-tundra occurred in the south and in the north, respectively, while during GIs Mediterranean forest developed in the south and temperate-cool forests further north. Interestingly, Mediterranean forest below 40°N was strongly developed during periods of minima in precession that exacerbated the seasonality of climate, and particularly winter precipitation. The dominance in the south of semi-desert and in the north of grasslands during HS 4 would have created an ecological barrier between the last Neanderthal living in the south of Iberia and the *H. sapiens* arriving in western Europe. This barrier allowed Neanderthals to avoid competition with *H. sapiens* for the same ecological niches and survive for several millennia in this region until the warming and wetting of GI 8 facilitated the advance of *H. sapiens.*
4. The instability of the EIS certainly provoked the particularly high-frequency environmental changes of the Last Glacial period in Europe. Steppe expansions associated with human demography increase as a result of the increases in animal biomass are observed during GSs. These repeated increases in human population during GSs may have triggered the succession of innovations and the emergence of the different cultures marking the Upper Paleolithic. If this correlation between climate and demography is confirmed in the future, when more dated sites will be available, it will be possible to argue that some of the key mechanisms that have governed the relationship between climate and prehistoric hunting peoples have been identified.

## Data Availability

The data that support the findings of this study are available from online sources associated with the references cited in the paper.

## Supplementary material

For supplementary material accompanying this paper visit https://doi.org/10.1017/ehs.2020.56. click here to view supplementary material
